# Universal Germline Genetic Testing in a Precision Oncology and Rare Cancer Clinic: Implementation and Outcomes

**DOI:** 10.1200/OA-25-00226

**Published:** 2026-06-02

**Authors:** Phebe Lemert, Hui-Zi Chen, Aditya Shreenivas, Kendra Eaves, Razelle Kurzrock

**Affiliations:** 1 Department of Obstetrics and Gynecology, Medical College of Wisconsin, Milwaukee, WI; 2 Insitute for Health and Humanity, Medical College of Wisconsin, Milwaukee, WI; 3 Division of Hematology and Oncology, Department of Medicine, Medical College of Wisconsin, Milwaukee, WI; 4 Discovery and Developmental Therapeutics Research Program, Medical College of Wisconsin Cancer Center, Milwaukee, WI; 5 Linda T. and John A. Mellowes Center for Genomic Sciences and Precision Medicine, Medical College of Wisconsin, Milwaukee, WI; 6 Department of Medical Oncology and Therapeutics Research, City of Hope Duarte Cancer Center, Duarte, CA

## Abstract

**PURPOSE:**

Current National Comprehensive Cancer Network (NCCN) guidelines for germline testing are complex and often miss patients with cancer susceptibility. We evaluated results of universal germline genetic testing in a Precision Medicine and Rare Cancer clinic, focusing on the prevalence of pathogenic/likely pathogenic (P/LP) variants and the proportion of affected patients who would have been missed by NCCN guidelines, comparing patients with rare and common cancer.

**METHODS:**

All consenting patients with cancer seen at Froedtert Hospital’s Precision Oncology and Rare Cancer clinic (June 2023 to January 2025; N = 120 patients; 55, common cancers, 65 rare cancers), who had not previously had germline testing, met with a clinic-embedded genetic counselor and underwent germline testing. Key variables included cancer type (rare *v* common), germline test results (penetrance/clinical actionability), and whether patients met NCCN guidelines for testing.

**RESULTS:**

The positive rate for P/LP germline variants was 15% (n = 18; 13%, patients with common cancer; 17%, patients with rare cancer). Notably, 29% and 64%, respectively, of patients with positive common and rare cancer did not meet NCCN guidelines at the time of testing. Among patients with common and rare cancer with positive results, 83% and 42% of P/LP variants were in high/moderate penetrance genes (eg, *BRCA2, PMS2, SDHB, ATM, CHEK2*).

**CONCLUSION:**

Universal germline testing identified a substantial number of patients with clinically valuable germline variants, many of whom would be missed by NCCN guidelines, exposing guideline-based testing limitations. Overall, 50% of patients positive for germline variants did not meet NCCN testing criteria. A clinic-embedded genetic counselor facilitated testing and post-test counseling as part of a comprehensive cancer management approach. These findings emphasize the need for inclusive testing strategies to better identify cancer predisposition, including in rare cancers.

## INTRODUCTION

Although most cancer is sporadic, approximately 5%–10% is hereditary.^1^ Germline testing can provide insights into hereditary cancer risk for patients and their families. The National Comprehensive Cancer Network (NCCN) provides eligibility criteria for germline testing, which are commonly used by both private insurers and Medicare.^2^ However, these guidelines are complex, have low rates of adherence, and miss patients who carry a pathogenic or likely pathogenic variant.^3–8^ To improve access to this valuable information, universal germline testing—where all patients with cancer are offered testing—has been proposed as a solution.^2,9^

We examine universal germline testing outcomes and related variables in a Precision Medicine and Rare Cancer clinic. Overall, we found that an important subset of patients with both rare cancers (defined as <6 per 100,000 persons)^10^ and common cancers had pathogenic/likely pathogenic (P/LP) germline variants despite not meeting NCCN criteria for germline testing.

## METHODS

The treating oncologist offered patients seen in Froedtert Hospital’s Precision Oncology and Rare Cancer clinic (June 27, 2023-January 28, 2025) germline genetic testing during their initial visit to the clinic, if it was not previously done. If patients indicated willingness to undergo testing, they met with a cancer genetic counselor embedded in the clinic for pretest counseling. Verbal consent to undergo germline testing was documented in the genetic counseling note.

The patients underwent a pan-cancer multigene panel, completed by a commercially available Clinical Laboratory Improvement Amendments-certified/clinical-grade laboratory (Data Supplemental, Table S1 for panels). Testing was performed using either saliva, buccal sample, or blood. Variants were classified by the laboratory as P/LP (grouped as positive for this study), variant of unknown significance, or negative (benign), in accordance with American College of Medical Genetics and Genomics variant classification guidelines.^11^ The clinic’s genetic counselor subsequently completed results review, disclosure, and post-test counseling. If a patient died before results disclosure and post-test counseling, results were disclosed to the patient’s estate.

This study follows the guidelines of the Medical College of Wisconsin institutional review board-approved study PREDICT-MCW (ClinicalTrials.gov identifier: NCT05802069) and any investigational interventions for which the patients provided consent.

Patient variables of interest included cancer type, germline genetic test result, and whether they met NCCN Guidelines for germline genetic testing. Cancer type was further characterized using the European definition of rare cancer, <6 cases per 100,000 persons.^10^ P/LP germline variants were further characterized as high (relative risk [RR] >4), moderate (RR = 2–4), or low (RR <2) penetrance, recessive, or uncertain clinical actionability based on disease risks and prior modeling.^12–14^ Additionally, P/LP germline variants with flagged concern for clonal hematopoiesis of indeterminate potential were characterized as uncertain clinical actionability.

## RESULTS

Of 206 patients seen during the period, 120 consented to germline genetic testing; only one patient who was approached by a genetic counselor did not consent. Other reasons why patients did not pursue germline testing include having previously completed germline testing, declined interest to see the genetic counselor, or agreed to see the genetic counselor on a return visit, but did not return to the clinic.

Of 120 patients who underwent germline testing in the Precision Oncology and Rare Cancer clinic during the designated period, 55 patients had common cancers and 65 patients had rare cancers (Table 1). The most frequent rare cancer types were cancer of unknown primary, sarcoma, brain, cholangiocarcinoma, and testicular cancer. Among common cancers, breast, lung, colorectal, pancreatic, and prostate cancer were most prevalent (Data Supplement, Table S2).

**FIG 1.**
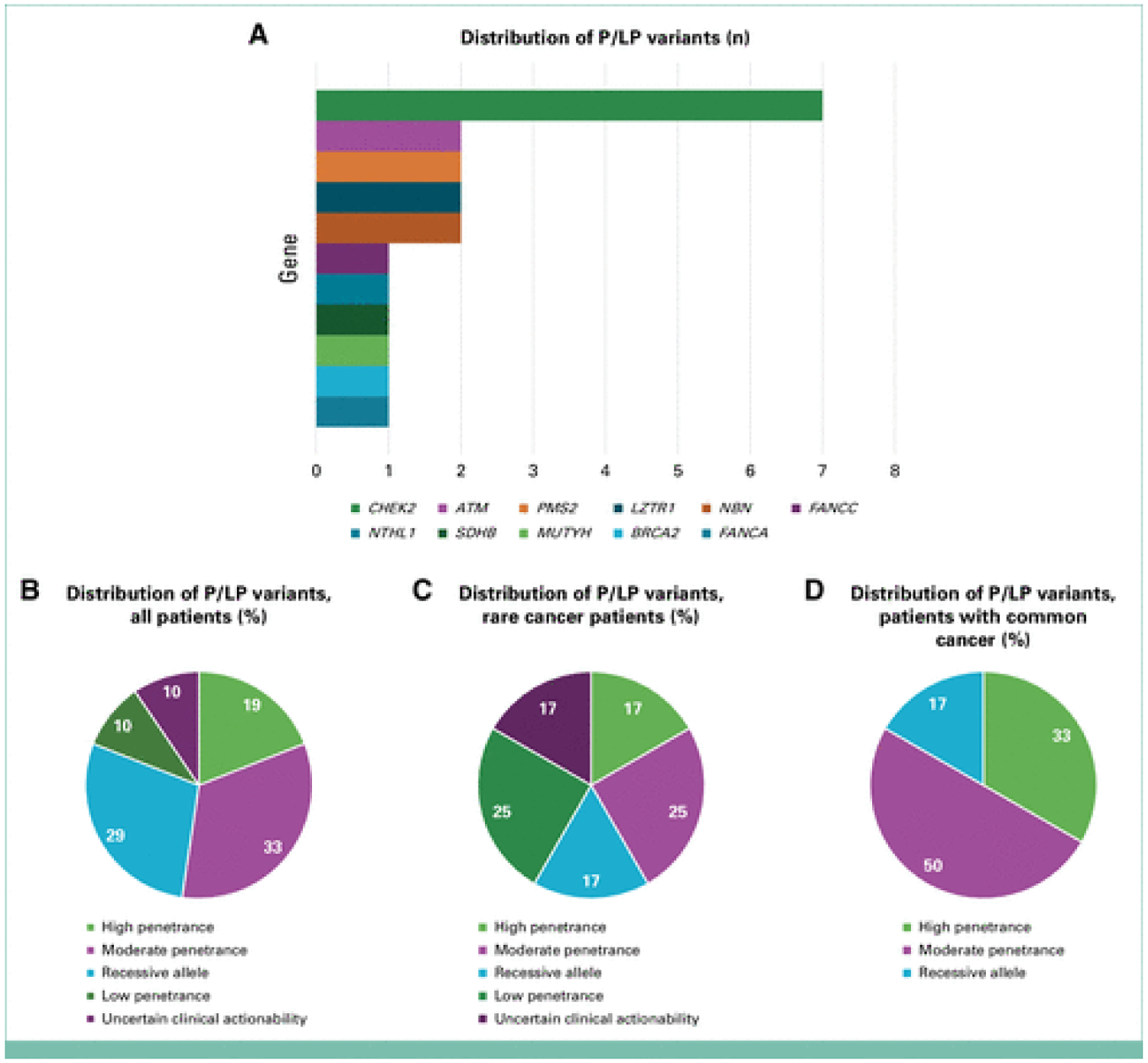
(A) Distribution of genes harboring P/LP variants among 120 patients in the Precision Medicine and Rare Cancer Clinic. Counts reflect the total number of variants; patients with more than one variant contribute multiple counts. (B) Distribution of the types of P/LP variants among all 120 patients. Categories include high penetrance, moderate penetrance, recessive alleles, low penetrance, and uncertain clinical actionability. (C) Distribution of P/LP variant types among the 11 rare cancer patients with positive germline findings. (D) Distribution of P/LP variant types among the seven patients with common cancer with positive germline findings. P/LP, pathogenic/likely pathogenic.

P/LP germline variants were frequent in both common and rare cancers, including in patients who did not meet NCCN criteria for germline testing (Figs 1A–1D, Table 1). Overall, 18 of 120 patients (15%) had a P/LP variant (Fig 1B). Of 55 patients with a common cancer, 13% (n = 7) were found to harbor at least one P/LP variant. This included patients with the following cancers: colorectal, prostate, endometrial, and thyroid; 29% of these positive patients with common cancers did not meet NCCN guidelines at the time of testing (Table 1). Of 65 patients with a rare cancer, 17% (n = 11) were found to harbor at least one P/LP variant. This included patients with the following cancers: squamous cell carcinoma of the buccal cavity, papillary renal cell carcinoma, sarcoma, brain, and cancer of unknown primary. Notably, 64% of these positive patients with rare cancers did not meet NCCN guidelines at the time of testing (Table 1). These findings suggest that current NCCN guidelines may miss a subset of patients with clinically valuable germline variants, especially in patients with rare cancers. In this context, clinical value refers to the information a P/LP variant may provide, including implications for medical management, targeted treatment options, and/or the potential for cascade testing in at-risk relatives. To further illustrate phenotype, pedigrees are provided for four patients with P/LP variants—two with common cancers and two with rare cancers—highlighting the range of personal and family histories (Data Supplement, Figs S1A–S1D).

The spectrum of P/LP germline variants identified in this cohort underscores the differences in penetrance and potential clinical value of germline testing between common and rare cancers. *CHEK2, ATM, PMS2, LZTR1, NBN, FANCC, NTHL1, SDHB, MUTYH, BRCA2*, and *FANCA* were identified to have P/LP variants. The gene most frequently detected with a P/LP variant was *CHEK2* (n = 7; Fig 1A). When characterizing by penetrance, 52% of positive results for all patients were in a high/moderate penetrance gene (*BRCA2, PMS2, SDHB*; *ATM, CHEK2*; Fig 1B). Positive results in high/moderate penetrance genes are particularly important because they can guide precision oncology approaches, such as poly (ADP-ribose) polymerase inhibitor and immunotherapy use, and support eligibility for tumor-agnostic clinical trials.^16,18,30,32^ Additionally, screening and potentially other risk-reducing management recommendations are available by NCCN and other professional organizations. Additionally, 42% of positive results were in a high/moderate penetrance gene for patients with rare cancer (Fig 1C). However, for patients with common cancers, 83% of positive results were in a high/moderate penetrance gene (Fig 1D). Of note, only two patients with a positive result in a high/moderate penetrance gene did not meet NCCN guidelines (Table 1). Overall, these findings highlight the potential for germline testing to uncover clinically valuable variants in patients who may not meet current guideline-based criteria, particularly in those with common cancers. It is also important to note that 8 of 21 variants detected were in a gene with low penetrance (*LZTR1*) or a recessively acting gene (*MUTYH, NBN, NTHL1, FANCA, FANCC*; Table 1). Although not immediately actionable, these genes provide value for cascade testing, especially for relatives of reproductive age. Furthermore, because these genes are integral to genomic maintenance and DNA repair pathways, they represent potential targets for future precision oncology therapies.^33,34^

## DISCUSSION

This study demonstrates the clinical value of universal germline testing across both common and rare cancers. In this context, clinical value refers to the information a P/LP variant may provide, including implications for medical management, targeted treatment options, and/or the potential for cascade testing in at-risk relatives. A substantial proportion of patients with P/LP variants in the Rare Cancer and Precision Medicine clinic would have been missed by guideline-based criteria. An overall positive rate of 15% in our cohort aligns with prior studies on universal germline testing, indicating that hereditary risk is not un-common in patients with cancer, regardless of age, family history, or cancer type.^2,9–12^

Current testing guidelines underestimate the prevalence of P/LP variants in patients with both rare and common cancers, according to our data. Notably, we observed a germline positive rate of 17% in our patients with rare cancers, with diagnoses including squamous cell carcinoma of the buccal cavity, papillary renal cell carcinoma, sarcoma, brain, and cancer of unknown primary; the rate in common cancers was 13%, and encompassed patients with colorectal, prostate, endometrial, and thyroid cancer. Importantly, 64% of patients with positive rare cancer and 29% of patients with positive common cancer did not meet NCCN criteria for germline testing at the time of initial visit to clinic. Our overall observations are consistent with prior literature indicating that NCCN guidelines miss a substantial portion of patients with a germline variant conferring cancer susceptibility.^2,3,9–13,17^ Additionally, our observations with patients with rare cancer are also in line with prior literature indicating that germline variants are indeed present in this population and information about them may offer clinical value.^9,15^ It is important to emphasize that patients with rare cancer are historically understudied and frequently lack relevant clinical guidelines. These findings overall emphasize the limitations of guideline-based testing and support universal germline testing as a more inclusive strategy.

The spectrum of identified germline variants also supports the clinical value of testing. Over half of P/LP variants were in a high/moderate penetrance gene, including *BRCA2, PMS2, SDHB, ATM*, and *CHEK2*, which can guide precision oncology treatment decisions, support eligibility for tumor agnostic clinical trials, and carry management recommendations for screening and/or risk reduction. Overall, 83% of P/LP variants in patients with common cancers were in a high/moderate penetrance gene. This aligns with prior research indicating universal germline testing may be particularly valuable for common cancers, such as breast, prostate, and colorectal.^19–23^ By contrast, the value of a P/LP variant was more limited for patients with rare cancers, but 42% were still in a high/moderate penetrance gene. A portion of P/LP variants detected were in recessive or low penetrance genes, including *MUTYH, NBN, NTHL1, FANCA, FANCC*, and *LZTR1*. These genes may help inform future targeted therapies and are relevant for reproductive-age family members, as cascade testing can guide family-planning decisions.

A unique aspect of this study is the integration of a clinic-embedded genetic counselor, which distinguishes it from most prior pan-cancer universal germline testing research. Clinic-embedded genetic counselors have been associated with improved and timely access to genetic counseling services and germline testing across several cancer types, including breast, pancreatic, or gynecologic cancer, and our study extends this model to a broader cancer population.^24–26^ Embedded genetic counselors may be especially beneficial when working with patients with rare cancer. They play a critical role in facilitating informed consent, interpreting results accurately, providing post-test support to the patient and their family, and minimizing redundant testing—functions that are essential for the successful implementation of germline testing.

Our study is limited by being at a single institution and having relatively small sample size. However, our data are consistent with other publications including specific rare cancer types such as sarcomas.^27^ Next steps include leveraging larger cohorts and longitudinal assessment of patient outcomes. Overall, our data support universal germline testing as a strategy to uncover clinically valuable information, perhaps especially in rare cancers. Rare cancers are individually un-common, but altogether compromise approximately 20% of the US cancer burden.^9 ,28–31^ Notably, 64% and 29% of patients with rare and common cancer, respectively, with positive results did not meet NCCN testing criteria, underscoring the need for a more universal testing approach. Our observations are consistent with others, suggesting that germline findings are relatively frequent in patients with cancer, with 15% of our patients showing such alterations. Notably, a clinic-embedded genetic counselor assisted in providing comprehensive management, which included testing for and counseling regarding hereditary cancer risks.

## Supplementary Material

Data Supplement

## Figures and Tables

**TABLE 1.** Germline P/LP Variants in the Precision Medicine and Rare Cancer Clinic (n = 21 total P/LP variants detected; n = 18 of 120 total patients; includes 65 patients with rare cancers)

Patient Group	Total Patients Tested (n)	Total Positive Patients, No. (%)	Total Negative Patients, No. (%)	Total VUS Patients, No. (%)	Patients Meeting NCCN Guidelines, All Results, No. (%)	Patients Who Did Not Meet NCCN Guidelines, Positive Results, No. (%)	P/LP Variants in a High/Moderate Penetrance Gene, (%)	Patients Who Did Not Meet NCCN Guidelines, But Had Positive Results in a High/Moderate Penetrance Gene, No. (%)
Patients with rare cancers	65	11 (17)	31 (48)	23 (35)	25 (38)	7 (64)	42	1 (20)
Patients with common cancers	55	7 (13)	31 (56)	17 (31)	31 (56)	2 (29)	83	1 (20)
Total patients	120	18 (15)	62 (52)	40 (33)	56 (47)	9 (50)	52	2 (20)
Germline P/LP Variants by Gene and Cancer Type								
Gene	Total No. of P/LP variants (n)			Cancer Type (n)				Comment
*BRCA2*	1			Colorectal (1)				High penetrance
*PMS2*	2			Colorectal (1), squamous cell carcinoma of buccal cavity (1)				
*SDHB*	1			Brain (1)				
*ATM*	2			Prostate (1), thyroid (1)				Moderate penetrance
*CHEK2*	5			Cancer of unknown primary (1), papillary renal cell carcinoma (1), colorectal (1), brain (1), prostate (1)				
*LZTR1*	2			Cancer of unknown primary (2)				Low penetrance
*MUTYH*	1			Prostate (1)				Recessive allele (biallelic P/LP variant required for cancer risk)
*NBN*	2			Endometrial (1), Sarcoma (1)				
*NTHL1*	1			Cancer of unknown primary (1)				
*FANCA*	1			Colorectal (1)				
*FANCC*	1			Mesothelioma (1)				
*CHEK2* p.I157T	2			Sarcoma (1), cancer of unknown primary (1)				Uncertain clinical actionability
Total P/LP variants detected	21							

NOTE. Table summarizes total patients tested, number and percentage of positive, negative, and VUS results, NCCN-eligibility, penetrance categories, and distribution of P/LP variants by gene and cancer type.

Abbreviations: NCCN, National Comprehensive Cancer Network; P/LP, pathogenic/likely pathogenic; VUS, variant of unknown significance.
