# Supplementary material for: Universal Germline Genetic Testing in a Precision Oncology and Rare Cancer Clinic: Implementation and Outcomes
Source: JCO Oncol Adv. Author manuscript; Available in PMC 2026 Jul 9. (PMC13344466; doi:10.1200/OA-25-00226)
Supplement: Data Supplement [file NIHMS2191274-supplement-Data_Supplement.pdf]

Supplemental Table 1. Germline Panels Utilized

Lab	Panel Name	Total Genes on Panel (n)	Total Patients Tested (n)
Tempus	Tempus xG+ Extended Hereditary Cancers Panel (powered by GeneDx)	88	59
Tempus	Tempus xG Common Hereditary Cancers Panel (powered by GeneDx)	52	2
Tempus	Tempus xG+ CancerNext-Expanded (powered by Ambry Genetics)	77	41
Tempus	Tempus xG+ CancerNext-Expanded (powered by Ambry Genetics)	76	1
Tempus	Tempus xG+ CancerNext-Expanded +RNAinsight (powered by Ambry Genetics)	77	2
Tempus	Tempus xG+ CancerNext-Expanded +RNAinsight (powered by Ambry Genetics)	76	2
Tempus	Tempus xG CancerNext (powered by Ambry Genetics)	36	3
Ambry	CustomNext +RNAinsight	62	5
Invitae	Custom Cancer + Sarcoma Panel	75	1
Invitae	Custom Cancer + Sarcoma + Preliminary Breast Panel	83	1
Invitae	Custom Cancer Panel	66	1
Invitae	Custom Cancer Panel	63	1
Invitae	Custom Cancer + RASopathies and Noonan Spectrum Disorders Panel	102	1

Supplemental Table 2. Details of Germline Variants by Cancer Type

	Common Cancers					
	Total Patients Tested (n)	Total Patients meeting NCCN guidelines (n)	Percent Positive (%)	Percent of patients who did not meet NCCN guidelines, positive results (%)	Percent Negative (%)	Percent VUS (%)
**Breast**	10	8	0	NA	60	40
**Lung**	10	5	0	NA	60	40
**Colorectal**	8	1	38	33	25	38
**Pancreatic**	7	7	0	NA	86	14
**Prostate**	6	6	33	0	67	0
**Hepatocellular carcinoma**	4	0	0	NA	75	25
**Thyroid**	3	1	33	0	0	67
**Melanoma**	3	1	0	NA	67	33
**Urothelial**	3	1	0	NA	0	100
**Endometrial**	3	0	33	100	67	0
**Clear cell renal cell carcinoma**	2	0	0	NA	50	50
**Gastric**	1	1	0	NA	0	100
	**Rare Cancers**					
	Number of Patients Tested	Number of Patients that met NCCN	% Positive	% Positive & did not meet NCCN	% Negative	% VUS
**Cancer of unknown primary**	22	6	18	100	50	32
**Sarcoma**	8	2	25	100	25	50
**Brain**	6	6	33	0	33	33
**Cholangiocarcinoma**	4	2	0	NA	50	50
**Testicular**	2	0	0	NA	100	0
**Mesothelioma**	2	1	50	100	0	50

**Pancreatic neuroendocrine tumor**	1	1	0	NA	0	100
**Basal Cell Carcinoma**	1	0	0	NA	100	0
**Squamous cell carcinoma of buccal cavity**	1	1	100	0	0	0
**Appendiceal**	1	0	0	NA	100	0
**High Grade Immature Teratoma of Ovary**	1	0	0	NA	100	0
**Thymoma**	1	0	0	NA	100	0
**Sinonasal**	1	0	0	NA	100	0
**Ependymoma**	1	0	0	NA	100	0
**Squamous Cell Carcinoma of Tonsil**	1	1	0	NA	100	0
**Salivary duct carcinoma**	1	1	0	NA	0	100
**Uveal Melanoma**	1	1	0	NA	100	0
**Gallbladder**	1	1	0	NA	100	0
**Endometrial carcinosarcoma**	1	1	0	NA	0	100
**Langerhans cell histiocytosis**	1	1	0	NA	0	100
**Neuroendocrine carcinoma**	1	0	0	NA	0	100
**Papillary renal cell carcinoma**	1	1	100	0	0	0

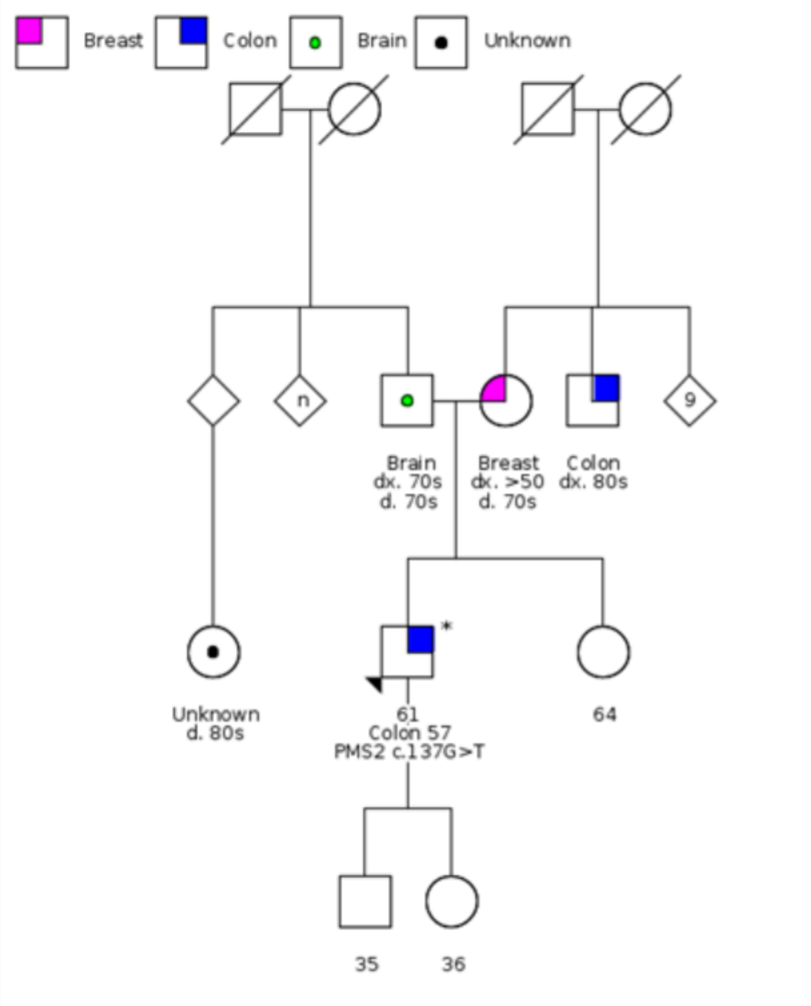

Supplemental Figure 1, Panel A: The pedigree illustrates the personal and family history of a patient with a *PMS2* c.137G>T pathogenic variant identified through Tempus xG+ germline testing (88 genes analyzed). The patient was initially seen in clinic for metastatic, MMR-intact colorectal cancer, initially diagnosed at 57. Prior to germline testing, neither Guardant360 nor Tempus xT somatic testing detected the *PMS2* variant and reported microsatellite stability. Additionally, the patient did not meet NCCN guidelines at the time of testing.

Standard pedigree symbols are utilized and family history is based on patient report. Squares indicate males, circles indicate females, and diamonds indicate sex not otherwise specified. Within shapes, “n” indicates an unspecified number of individuals, while a number designates that number of individuals (e.g., 9 = 9 individuals). Affected individuals are shaded per the above key. Underneath each individual, current age, age at diagnosis (“dx.”), age at death (“d.”), and other relevant history (e.g., genetic test result) are noted. A slash denotes deceased individuals, an asterisk indicates those with genetic testing, and an arrow identifies the proband.

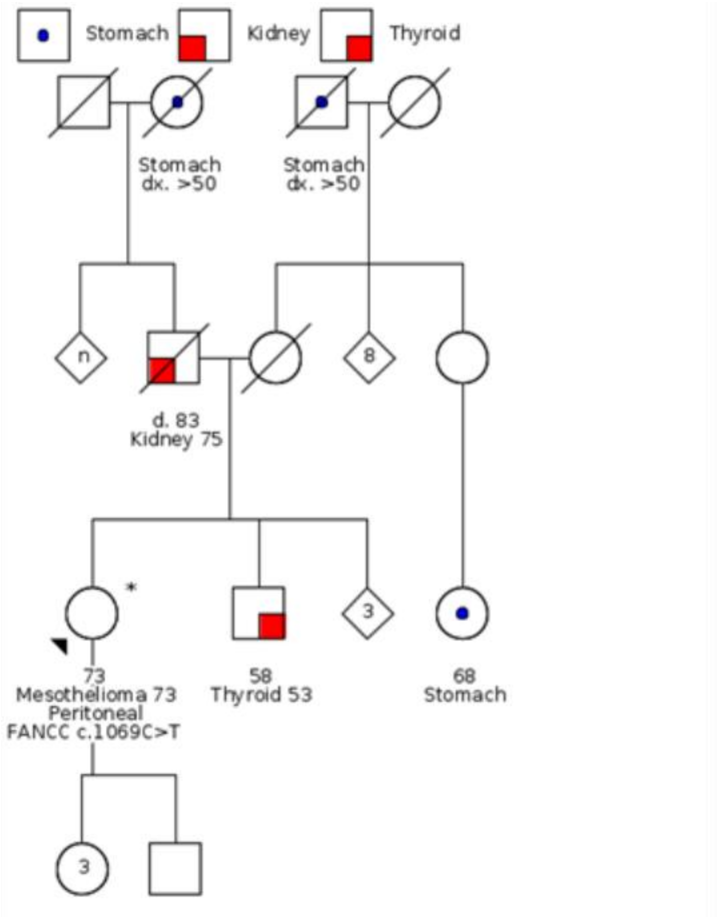

Supplemental Figure 1, Panel B: The pedigree illustrates the personal and family history of a patient with a *FANCC* c.1069C>T pathogenic variant identified through Tempus xG+ germline testing (77 genes analyzed). The patient was initially seen in clinic for peritoneal mesothelioma, diagnosed at 73. The patient did not meet NCCN guidelines at the time of testing.

Standard pedigree symbols are utilized and family history is based on patient report. Squares indicate males, circles indicate females, and diamonds indicate sex not otherwise specified. Within shapes, "n" indicates an unspecified number of individuals, while a number designates that number of individuals (e.g., 8 = 8 individuals). Affected individuals are shaded per the above key. Underneath each individual, current age, age at diagnosis ("dx."), age at death ("d."), and other relevant history (e.g., genetic test result) are noted. A slash denotes deceased individuals, an asterisk indicates those with genetic testing, and an arrow identifies the proband.

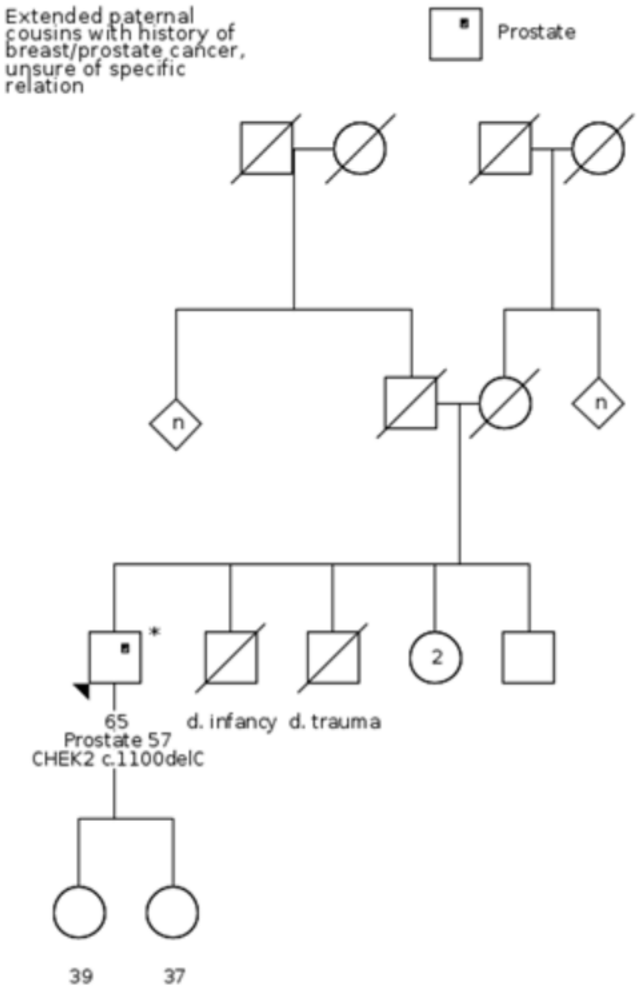

Supplemental Figure 1, Panel C: The pedigree illustrates the personal and family history of a patient with a *CHEK2* c.1100delC pathogenic variant identified through Tempus xG+ germline testing (76 genes analyzed). The patient was initially seen in clinic for metastatic castrate-resistant prostate cancer, diagnosed at 57. Prior to germline testing, both FoundationOne CDx and Guardant360 somatic testing identified the *CHEK2* variant. Additionally, the patient met NCCN guidelines at the time of testing.

Standard pedigree symbols are utilized and family history is based on patient report. Squares indicate males, circles indicate females, and diamonds indicate sex not otherwise specified. Within shapes, "n" indicates an unspecified number of individuals, while a number designates that number of individuals (e.g., 2 = 2 individuals). Affected individuals are shaded per the above key. Underneath each individual, current age, age at diagnosis ("dx."), age at death ("d."), and other relevant history (e.g., genetic test result) are noted. A slash denotes deceased individuals, an asterisk indicates those with genetic testing, and an arrow identifies the proband.

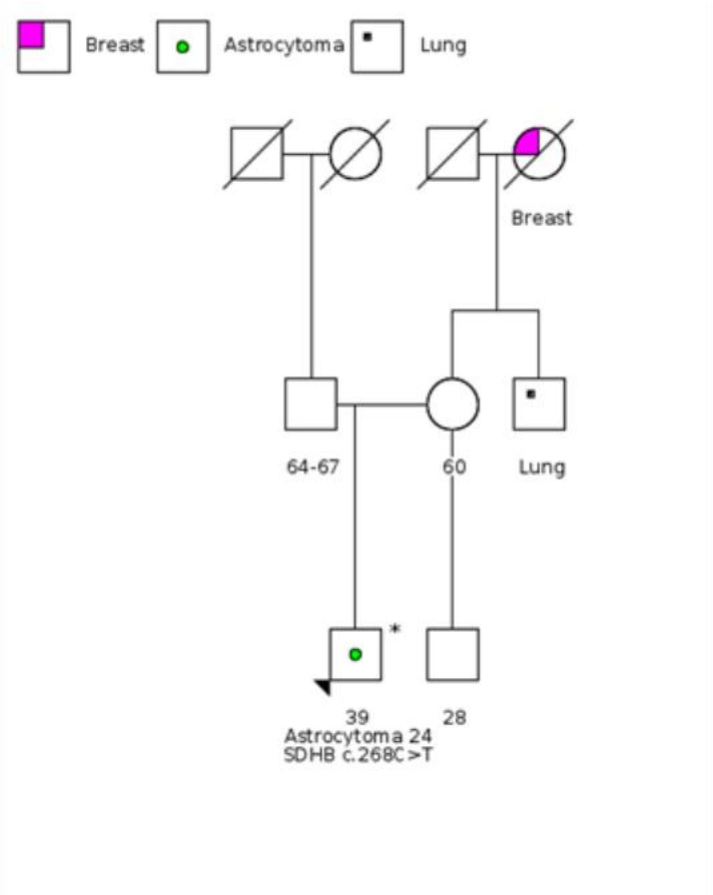

Supplemental Figure 1, Panel D: The pedigree illustrates the personal and family history of a patient with a *SDHB* c.268C>T pathogenic variant identified through Tempus xG+ germline testing (77 genes analyzed). The patient was treated for WHO Grade IV astrocytoma, IDH-mutated, initially diagnosed at age 24. Prior to germline testing, the patient's Tempus xT somatic testing identified the *SDHB* variant. Additionally, the patient met NCCN guidelines at the time of testing.

Standard pedigree symbols are utilized and family history is based on patient report. Squares indicate males, circles indicate females, and diamonds indicate sex not otherwise specified. Affected individuals are shaded per the above key. Underneath each individual, current age, age at diagnosis, and other relevant history (e.g., genetic test result) are noted. A slash denotes deceased individuals, an asterisk indicates those with genetic testing, and an arrow identifies the proband.
